# Supplementary material for: Mitochondrial HMG-Box Containing Proteins: From Biochemical Properties to the Roles in Human Diseases
Source: Biomolecules. 2020 Aug 16;10(8):1193. doi: 10.3390/biom10081193 (PMC7463775; doi:10.3390/biom10081193)
Supplement: Supplementary file 1 [file biomolecules-10-01193-s001.zip › Supplementary_Figure_S1.pdf]

Vozáriková *et al*.
Supplementary **Figure S1**.

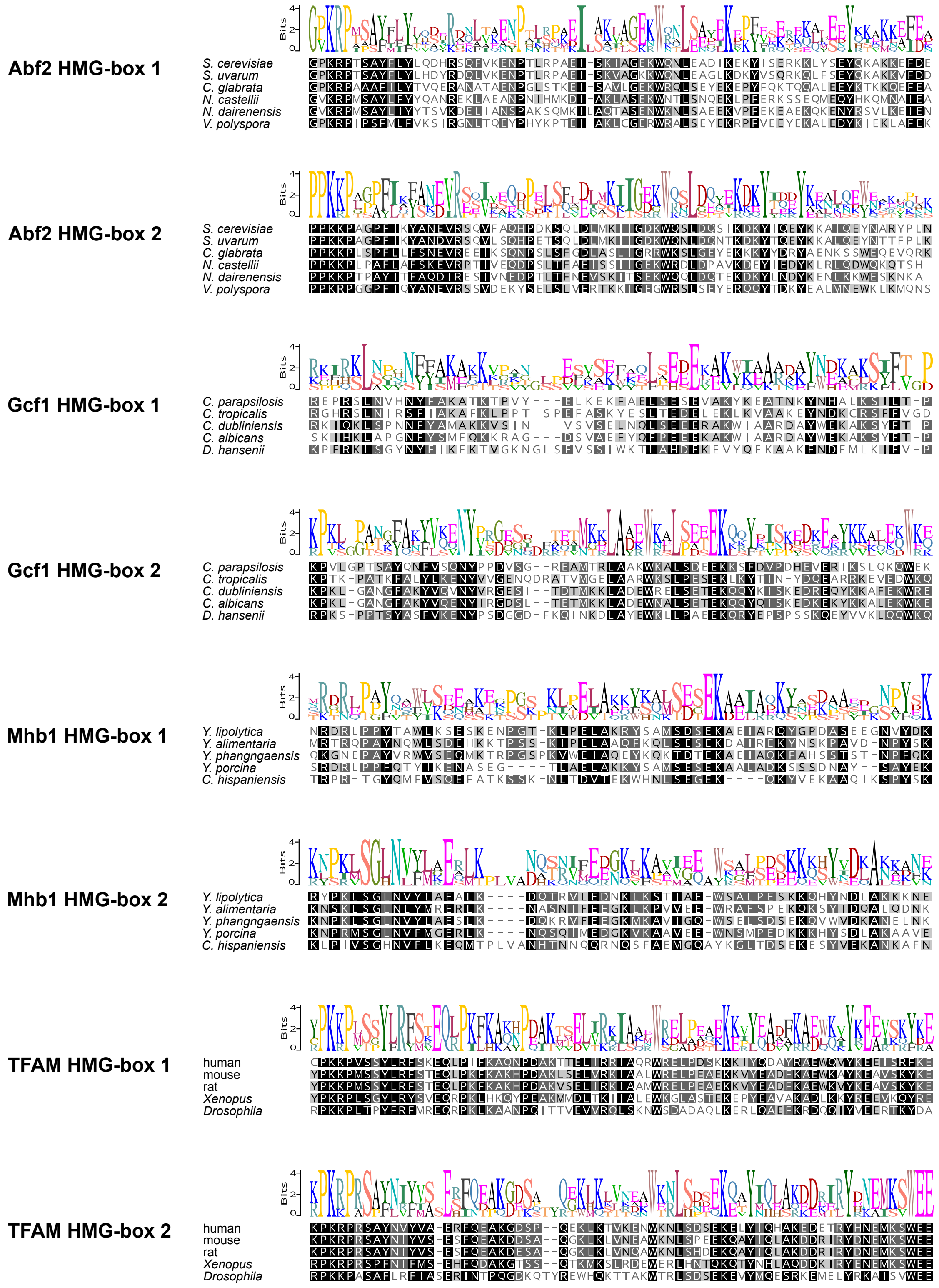

Figure S1. Amino-acid sequence alignment of HMG-box domains 1 and 2 from yeast (Abf2, Gcf1, Mhb1) and human (TFAM) mtHMG proteins. The HMG-box domains of the given species were predicted using InterProScan [1], aligned using MAFFT v. 7.450 [2] and the sequence logos were created using Geneious v. 11.1.5 (Biomatters).

1. Jones, P.; Binns, D.; Chang, H.-Y.; Fraser, M.; Li, W.; Mcanulla, C.; Mcwilliam, H.; Maslen, J.; Mitchell, A.; Nuka, G.; et al. InterProScan 5: genome-scale protein function classification. *Bioinformatics* **2014**, *30*, 1236–1240, doi:10.1093/bioinformatics/btu031.
2. Katoh, K.; Standley, D.M. MAFFT multiple sequence alignment software version 7: Improvements in performance and usability. *Mol. Biol. Evol.* **2013**, *30*, 772–780, doi:10.1093/molbev/mst010.
